# Supplementary figures and images for: Evidence based targeting of districts for active surveillance of skin-related neglected tropical diseases in Ghana
Source: PLOS Glob Public Health. 2026 Mar 27;6(3):e0006074. doi: 10.1371/journal.pgph.0006074 (PMC13028356; doi:10.1371/journal.pgph.0006074)

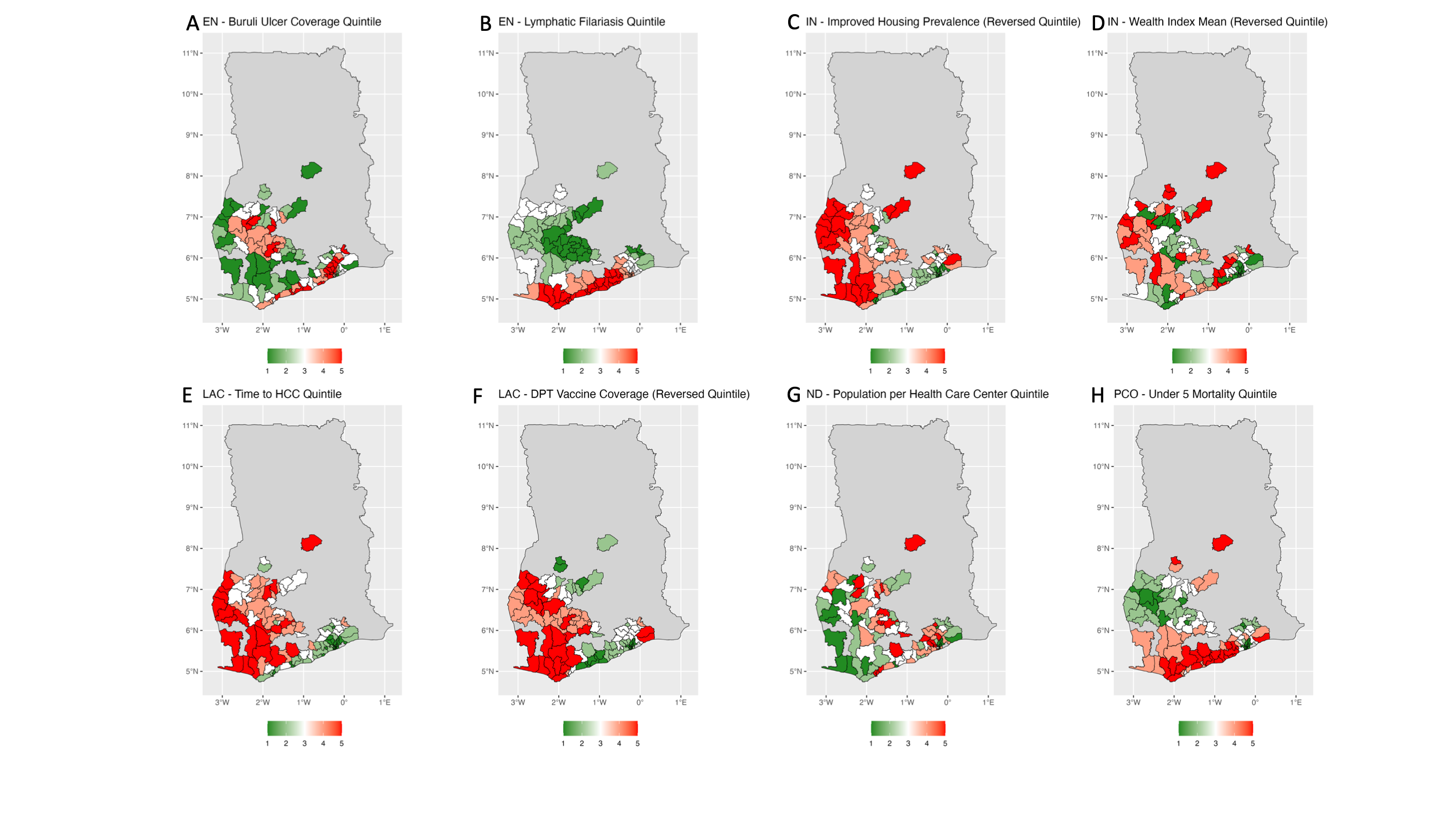

Supplement: S1 Fig — (TIFF) [file pgph.0006074.s001.tiff]
