# Supplementary material for: Evidence based targeting of districts for active surveillance of skin-related neglected tropical diseases in Ghana
Source: PLOS Glob Public Health. 2026 Mar 27;6(3):e0006074. doi: 10.1371/journal.pgph.0006074 (PMC13028356; doi:10.1371/journal.pgph.0006074)
Supplement: Attachment — Submitted filename: Response_to_Reviewers.docx [file pgph.0006074.s003.docx]

Dear Jianhong Zhou,

We would like to thank you and the reviewer for your constructive feedback on our manuscript. We have revised the text in line with all comments received, with changes highlighted in the tracked version of the manuscript. Below, we provide a detailed, point-by-point response to each comment.

Comment 1: As a clinician I am not familiar with many of the concepts in this manuscript and so found it rather hard going to read and review. If your target audience is aiming at professionals familiar with working with geographic data then no modification is required, but if you wish to engage the wider Skin NTD community to understand this proposed method and concept it might be worth revising the manuscript with the less geographic data aware individuals in mind.

Response: We have revised the manuscript to make it more accessible to readers without a GIS or geostatistical background. This includes:
- Adding plain-language explanations of technical terms at first mention (e.g., raster files, shapefiles, quintiles).
- Inserting a new glossary of technical terms at the end of the manuscript.
- Rewriting several sections in the Abstract, Methods, and Discussion to include clear, step-by-step explanations of the workflow.
- Clarifying that the interactive dashboard requires no GIS or programming skills to use.

Comment 2: What is a rapid literature review?

Response: We have added a definition of a rapid literature review at first mention and cited the paper suggested by the reviewer (Smela et al., 2023).

Comment 3: You mention the use of Demographic and Health Surveys (DHS)... is the data uniform for each country?

Response: We have clarified that while DHS uses a standardised methodology, the exact indicators collected vary by country and survey year. We note that adaptation is needed when indicators are unavailable for certain countries.

Comment 4: Discussion – I have absolutely no knowledge of R or GIS so I have to take statements at face value.

Response: We have inserted a concise, non-technical summary of the workflow steps in the Discussion and added explanations throughout to ensure the logic and outputs can be followed without prior GIS or programming knowledge. We have also emphasised that the dashboard outputs are usable without any coding or GIS software, and that a glossary of key technical terms has been included to further aid accessibility.

## Editorial requests / minor changes

- All acronyms are now defined at first mention.
- Technical descriptions are now accompanied by plain-language summaries.
- The Discussion now includes both the current use for BU and LF and a clear explanation of adaptability for other settings.

We believe these revisions have substantially improved the clarity and accessibility of the manuscript while retaining the necessary technical detail for geospatial experts. We thank the reviewer again for their helpful suggestions and hope that the revised version addresses all concerns.

Sincerely,
William Jones-Warner
On behalf of all authors
