# Supplementary material for: Evidence based targeting of districts for active surveillance of skin-related neglected tropical diseases in Ghana
Source: PLOS Glob Public Health. 2026 Mar 27;6(3):e0006074. doi: 10.1371/journal.pgph.0006074 (PMC13028356; doi:10.1371/journal.pgph.0006074)
Supplement: Attachment — Submitted filename: Response_to_Reviewers_auresp_2.docx [file pgph.0006074.s004.docx]

Reviewer #3: I write to submit my review on the manuscript titled “Evidence-Based Targeting of Districts for Active Surveillance of Skin-Related Neglected Tropical Diseases in Ghana.”
The authors analyzed secondary data using an evidence consensus approach to summarize and synthesize a variety of spatial datasets.
Here are my comments

1. Who were the local experts whose opinions were integrated into the overall study design? Kindly provide details of selection criteria and their experiences in BU and LF research, case management and surveillance, diagnosis and treatment etc

- *We did not use local expert opinion but stated this in theory could be utilised in the absence of predicted suitability/reported cases. We have added a sentence to clarify this on lines 276-277:* “In the case of this report, predicted BU suitability and predicted LF antigenaemia were utilised.”

2. Kindly provide the predicted suitability range for BU. LF prevalence estimates will range from 0 to 100%. Was it the same for BU? Kindly state it. What is the definition of suitability range?

- *Thanks for spotting this omission. We have added the definition of suitability to the first mention of suitability after the introduction when it is used in the context of the data- Lines 276-279:* “In the case of this study, predicted BU suitability and predicted LF antigenaemia were utilised. In this context, ‘suitability’ denotes the availability of environmental conditions which best characterise locations with occurrence of Buruli ulcer cases, derived from a modelling study.”
- *Ranges of BU suitability have been included at the start of the results section line 444-446.*

3. Line 325-327 is a bit confuse. “These two metrics were used due to the shared endemicity in Ghana, using both could indicate the co-endemicity of other skin NTDs and the ready availability of the datasets”. Kindly revise the statement. I understand that the availability of data is one of the reasons only two metrics were considered in selecting the districts but how it is currently phrased is a bit confusing. This should be clearly stated. You may revise as follows: These two metrics were used due to the ready availability of the datasets and shared endemicity in Ghana; using both could indicate the co-endemicity of other skin NTDs.

*Thank you, we appreciate the suggested alternative phrasing which is much clearer.* 4. “We calculated mean values for each of the 260 districts(50)”. Mean values of what?

- *Thank you for spotting this. This was an artifact of a previous version and redundant within the current sentence. It has been removed.*

5. What informed the threshold of excluding districts in the bottom quintile (with predicted prevalence of 5% or less)? What would have been the implications on the statistical analyses if those districts with a predicted prevalence of 5% or less had been included in the analysis? These details and explanations must be provided in the manuscript

- *The threshold for excluding districts in the bottom quintile of predicted LF prevalence (≤5%) was set to prioritise areas where transmission is epidemiologically meaningful. Our approach is based on the principle of targeting resources to districts with the highest likelihood of undiagnosed cases, as reflected in the evidence consensus framework we applied.*
- *Including districts with very low predicted prevalence would add locations where the prevalence of LF morbidity is expected to be minimal. Excluding the bottom quintile ensured that our analysis focused on districts with sufficient predicted prevalence to meaningfully contribute to the identification of priority areas for intervention, consistent with the overall goal of targeting high-burden or high-risk districts for active case detection.*
- *We have added a sentence in the methods lines 347 – 351 justifying the approach*. “Districts in the bottom quintile of predicted LF prevalence (≤5%) were excluded to focus the analysis on locations with meaningful transmission potential. Including very low-prevalence districts would have increased the number of areas with minimal expected cases, reducing the sensitivity of the analysis to identify high-priority districts for active case detection.”
- *We have also added a short paragraph in the discussion addressing this decision lines 641 – 648. “Although WHO elimination guidelines for lymphatic filariasis use very low prevalence thresholds (for example, <1% microfilaria or <2% antigenaemia) to determine when transmission is no longer sustainable and mass drug administration can be stopped, districts with predicted prevalence of 5% or less were excluded from our analysis. At such low predicted prevalence, the likelihood of ongoing transmission and undiagnosed cases is minimal compared with higher-prevalence areas. Including these low-prevalence districts could have introduced additional noise, reducing the ability of the framework to detect and prioritise districts with meaningful transmission potential”*

6. Line 343-344: “We considered both of these indicators together to identify where BU and LF overlapped.” How did you combine the two indicators to identify overlap? Because line 345-348 only talks about how the two distributions were converted to quintiles separately

- *This paragraph has been amended to clarify that the groups were numbered 1-5 for BU and 2-5 for LF and these are added together to give a final score out of 10 for the domain. Lines 370 – 373::* “two representing the lowest quantile and five representing the quantile with the highest predicted LF prevalence). The scores across predicted BU suitability and predicted LF prevalence were combined to give a score out of ten (Table 3).”

7. Kindly include the implications for conducting single mean imputation using estimates from neighbouring districts instead of a more robust multiple imputation by chain equations

- *We have now added one paragraph to the discussion (see lines 708 - 720) outlining this limitation and advising that results for districts with imputed data be interpreted with caution. We also note that future applications of the framework could adopt multiple imputation techniques to better capture uncertainty and improve the robustness of district prioritisation:*
- *“In addition, the DHS dataset contained missing values for specific districts. To enable inclusion of all districts within the framework, we applied single mean imputation, replacing missing values with the mean of neighbouring districts. While this pragmatic approach allowed complete district coverage, it does not account for uncertainty in the imputed values and may underestimate true variability compared with more robust methods such as multiple imputation by chained equations. These limitations were acknowledged and considered acceptable given that individual indicators contribute only one component of a broader, multi-domain assessment rather than serving as standalone determinants. Future applications could incorporate multiple imputation approaches to better quantify uncertainty and strengthen the robustness of prioritisation”*

8. The DHS data sets are not powered at the district level and therefore wealth estimates will not be representative of the targeted districts. So, assessing vulnerability using DHS wealth quintiles at the district level is problematic as the sample is not representative of the targeted districts. DHS is stratified at the regional and rural, urban, and not powered at the district level, as the focus of the DHS is to generate national, regional and rural-urban stratification estimates. These limitations must be clearly highlighted and its implications on the current findings stated.

- *We have revised the Discussion to explicitly acknowledge that DHS data are not powered to produce district-level estimates and are stratified at the national, regional, and urban–rural levels. We now clearly state that district-level wealth indices derived from DHS should be interpreted as approximate indicators of relative socioeconomic vulnerability rather than representative district-level measures, particularly in districts with few sampled clusters (lines 697-706)*
- *“Several limitations related to data availability and representativeness should be considered when interpreting these findings. District-level wealth estimates derived from DHS household data should be interpreted with caution, as the DHS is designed to produce nationally and regionally representative estimates and is stratified by urban and rural residence rather than by district. Consequently, the relative wealth index values used here, derived from the Ghana DHS Phase 7 household dataset, should be viewed as approximate indicators of relative socioeconomic vulnerability rather than precise district-level estimates. Because the DHS sampling design is not intended to be representative at the district level, the households sampled within a given district may not fully reflect the socioeconomic characteristics of the district population as a whole, particularly in districts with few sampled clusters”*

9. What informed this definition? “We defined co-endemic districts as those with any area predicted suitable for BU, and with a mean predicted LF antigen prevalence of at least 5%.” Is it based on the literature or is a standard definition? Reference. It was chosen for the purposes of this study, then kindly provide justification.

- *The definition of co-endemic districts as those with any area predicted suitable for BU and a mean predicted LF antigen prevalence of at least 5% was chosen specifically for this study to prioritise districts likely to harbour substantial numbers of LF morbidity cases.. Line 434 – 441.*

“As the primary aim was to prioritise districts for integrated active case detection of skin NTDs, we restricted analyses to districts with plausible co-occurrence of BU and LF. Co-endemic districts were therefore defined as those with any area predicted suitable for BU and a mean predicted LF antigen prevalence of at least 5%. This threshold was chosen to exclude districts with low predicted LF prevalence where undiagnosed morbidity cases would be rare. This pragmatic definition was selected to focus the framework on districts most likely to benefit from integrated case detection rather than to provide a formal epidemiological definition of co-endemicity”

10. “Domain scores were then adjusted so that all domains carried equal weight, regardless of the number of component indicators” How did you adjust the domain score? Statistical method used for the adjustment?

- *We agree that this needs further detail. We adjusted domain scores using simple linear scaling so that each domain contributed equally to the total score.*
- *We have added more detail on lines 446- 450:* “To ensure that all domains carried equal weight, regardless of the number of component indicators, we adjusted the domain scores by linear scaling. Specifically, the scores from the "Availability of Health Services" and "Risk Factors for Poor Clinical Outcomes" domains were doubled to bring them to an equivalent maximum score of 10, aligning with the other domains

11. Major concern: There was no internal validation of the methodology to assess robustness in real application. Authors indicated that “Six districts, including Shama, Awutu Senya East, and Ekumfi, scored high for both BU and LF, making them priority areas for active BU detection”. What is the observed number of BU and LF cases in the six priority districts compared to the over 100 districts in Ghana? If the methodology can identify high-priority areas, then internal validity assessment of the approach should demonstrate that the true observed cases of BU and LF in the six districts exceed the true observed cases of BU and LF from other districts in Ghana. This detailed internal validity assessment was completely missing from the manuscript.

- We thank the reviewer for raising the issue of validation. We agree that evaluating whether districts prioritised by the framework correspond to higher observed counts of BU and LF is important in principle. However, we did not undertake such an analysis because routine surveillance data were not considered an appropriate standard for validation of true disease burden. Because the objective of the framework is to identify areas with the potential for undiagnosed disease rather than those with high reported case numbers, we judged routine surveillance unsuitable as a validation dataset and likely to bias the assessment towards - districts with stronger surveillance. We have now explicitly acknowledged this limitation in the Discussion. We also clarify that future validation should use community-based surveys or enhanced surveillance in prioritised districts to formally assess the framework’s performance in operational settings.
- We have added the following from lines 723 – 740; *“An important limitation of this study is the absence of validation using empirical data on BU and LF at the district level. Given that our primary aim was to identify areas with potential undiagnosed disease, robust validation would require data from population-based prevalence surveys across districts with different priority scores, which were beyond the scope of this study. Routine surveillance data were not considered an appropriate benchmark for validation of true disease burden, as they may be more reflective of surveillance sensitivity and health system access than underlying transmission intensity or unmet need. A validation study for LF demonstrated that routine surveillance underestimates lymphoedema by approximately 80% compared with community-based surveys (Simpson et al. 2025). Similar limitations apply to Buruli ulcer, where hospital- and clinic-based reporting is known to capture only a fraction of community cases due to delayed care-seeking, misdiagnosis, and limited access to diagnostic services (Mbabazi et al. 2010; Awuah et al. 2015). Targeted morbidity surveys for LF and active case-finding for BU represent an important direction for future work.”*
